# Hemodynamic effects of prophylactic amiodarone assessed by pressure–volume analysis in anesthetized female pigs in sinus rhythm—An exploratory study

**DOI:** 10.1371/journal.pone.0349600

**Published:** 2026-05-22

**Authors:** Laura Svanekjær, Emma Illum, Peter H. Frederiksen, Louise Linde, Anika Klein, Karoline Korsholm Jeppesen, Ann Banke, Sarah Holle, Lisette O. Jensen, Henrik Schmidt, Jens F. Lassen, Jacob E. Møller, Hanne B. Ravn

**Affiliations:** 1 Department of Cardiothoracic Anaesthesiology, Odense University Hospital, Odense, Denmark; 2 Department of Cardiology, Odense University Hospital, Odense, Denmark; 3 Department of Cardiology, Copenhagen University Hospital, Rigshospitalet, Copenhagen, Denmark; 4 Department of Clinical Research, University of Southern Denmark, Odense, Denmark; Scuola Superiore Sant’Anna, ITALY

## Abstract

**Background:**

Intravenous amiodarone is used for acute management of severe arrhythmias and can cause hypotension. However, amiodarone’s direct hemodynamic and myocardial effects during sinus rhythm are not well characterized.

**Methods:**

Twenty-five anesthetized female pigs (75–80 kg) received a 300 mg intravenous bolus amiodarone over 30 minutes. To assess hemodynamic effects, animals were instrumented with a left ventricular conductance catheter, a pulmonary artery catheter, a carotid artery Doppler flow probe, and a renal vein catheter. Data were recorded at baseline, after 15 and 30 minutes and presented as median with interquartile ranges.

**Results:**

Amiodarone reduced systolic and mean arterial blood pressure by 12% (p < 0.001) after 30 minutes infusion, primarily driven by 24% reduction in cardiac output due to combined deceased heart rate (87 (79; 99) beats per minute (bpm) to 72 (62; 81) bpm, p < 0.001) and stroke volume (69 (64; 79) mL to 64 (48; 73) mL, p = 0.004). End-systolic elastance (EES) decreased (0.54 (0.33; 0.63) mmHg/mL to 0.43 (0.29; 0.51) mmHg/mL, p < 0.001), while systemic vascular resistance and arterial elastance (EA) remained unchanged, resulting in an increased EA/EES ratio from 2.45 (2.01;3.53) to 3.26 (2.55; 4.30) (p < 0.001). This impaired cardiac function led to reduced carotid artery blood flow and renal perfusion pressure.

**Conclusion:**

Infusion of 300 mg amiodarone intravenously in anesthetized pigs in sinus rhythm impaired left ventricular contractility and caused ventriculo-arterial decoupling with increased EA/EES ratio. This led to reduced cardiac output, blood pressure, and reduced carotid- and renal blood flow.

## Background

Administration of intravenous amiodarone is a cornerstone in acute management of patients with hemodynamic compromise due to atrial or ventricular arrhythmias. In this setting, amiodarone can stabilize hemodynamics by treating arrhythmias that might otherwise cause hemodynamic instability [1–3]. Current ESC guidelines acknowledge intravenous amiodarone as an effective antiarrhythmic agent, while also emphasizing the risk of acute hypotension and bradycardia during administration [4–7]. Amiodarone has multichannel-blocking properties, encompassing Class I–IV effects according to the Vaughan Williams classification, but is classified as a type III antiarrhythmic agent. In addition to sodium, beta-adrenergic, and potassium channel blockade, amiodarone also inhibits L-type calcium channels [1,8]. This mechanism of action can prolong the action potential and refractory period leading to side effects such as bradycardia and reduced cardiac output (CO) [1]. Previous studies have demonstrated that amiodarone has an effect on the vascular circulation, leading to systemic hypotension [9,10], and decreased cardiac output (CO), due to reduced ventricular function [10–12]. It has been suggested that the potential vasodilatory effect was caused by the solvent vehicles used in intravenous formulations, such as polysorbate 80 and benzyl alcohol, but also amiodarone’s direct calcium channel blockade [2,10]. However, the underlying mechanisms remain incompletely understood during sinus rhythm, justifying further investigation.Previous studies investigating cardiogenic shock and mechanical circulatory support have primarily been conducted in translational large-animal porcine models. In these settings, amiodarone has been used prophylactically to prevent ventricular tachyarrhythmias during coronary artery catheterization and shock induction [13,14].The aim of this translational animal study was to examine the immediate effects of a prophylactic 30-minute amiodarone bolus infusion of 300 mg intravenously on hemodynamics, in terms of heart rate, cardiac output, and mean arterial pressure, as well as left ventricular function to explore the underlying pathophysiology in anesthetized pigs in sinus rhythm.

## Materials and methods

Twenty-five Danish female Landrace pigs (75–80 kg) from a single certified farmer on the island of Fyn in the Region of Southern Denmark were studied. The current study was part of a study protocol investigating mechanical circulatory support treatment during cardiogenic shock, and given the exploratory nature of this sub-study, no formal sample size calculation or power analysis was performed. Effect sizes and variability are reported, and all analyses are considered exploratory, so no correction for multiple comparisons were made. It met the expectation of the 3Rs framework, which aims to replace, reduce and refine animals in research.

The study was approved and conducted according to the Danish Animal Experiment Directorate rules and was approved under Study ID: 2006-15-00951. All animals were housed and provided according to Danish law regarding animal studies. Animals were anesthetized as described below and at the end of the experiment euthanized by surgical excision of the heart through a sternotomy [15].

### Animal preparation and instrumentation

On the day of the experiment, all animals were sedated with sevoflurane (MAC 4–6) and fentanyl (25–50 μg/kg/h) to alleviate any suffering, endotracheally intubated and mechanically ventilated as previously described [16]. During amiodarone infusion all ventilator settings were kept constant.

A conductance catheter (Ventricath 512 PV Loop Catheter, Millar Inc, TX, USA) was advanced into the left ventricle (LV) through an 8 Fr introducer sheath placed in the carotid artery. Systemic blood pressure was measured from this access. A pulmonary artery (PA) catheter was placed through a multi lumen sheath in the left external jugular vein (Edwards Lifesciences Corp, Irvine, CA, USA) and advanced to a PA for monitoring of PA pressure, mixed venous saturation (SvO_2_) and continuous thermodilution CO measurements (S1 Fig).

The left carotid artery was surgically exposed, and a Doppler flow probe (MEDISTIM SonoQ TTFM Probe, Emtec GmbH, Finning Germany) was used to measure carotid blood flow continuously. A Swan Ganz catheter was advanced from a femoral venous access into the right or left renal vein for continuous measurement of renal venous pressure (S1 Fig).

### Pressure-volume loop analysis

The conductance catheter was connected to a MPVS Ultra Pressure-Volume Loop (PVL) system (Millar Inc., TC, USA), and the Powerlab 16/35 system (ADInstruments, Dunedin) was used for data sampling and analysis in LabChart Pro (ADInstruments, Dunedin). Heart rate was recorded in LabChart Pro via continuous electrocardiogram monitoring.

Infusion of hypertonic saline was used to determine parallel wall conductance. The alpha calibration was calculated from the PA catheter thermodilution derived CO. For determination of the estimated ventricular volume at zero pressure (V_0_), an occlusion of the inferior vena cava was performed to reduce preload. V_0_ was calculated with a linear fit. V₀ was assumed to remain constant during amiodarone administration, which was initiated immediately after its determination, and in the absence of any additional interventions.

Variables obtained and calculated from the PVL analysis and additional invasive measurements are presented in S1 Fig.

### Intervention

After instrumentation and ensuring stable hemodynamic condition, baseline measurements were obtained, and an amiodarone bolus infusion (10 mg/mL) was administered (60 mL/hr) for 30 minutes (300 mg), based on the recommended loading dose in adult humans [4]. Data were collected before infusion, after 15, and 30 minutes of infusion for hemodynamics and conductance catheter derived data. No animals received vasopressors or inotropes during the amiodarone infusion.

### Statistics

Data are presented as medians with interquartile ranges. The Wilcoxon signed-rank test was used to test for differences between time points. The statistical significance level was set to < 0.05 for all analyses with no adjustment for multiple comparison due to the exploratory nature of the study.

All statistical analyses were conducted using R Statistical Software version 4.4.1 (R Core Team 2024).

## Results

**Fig 1:**
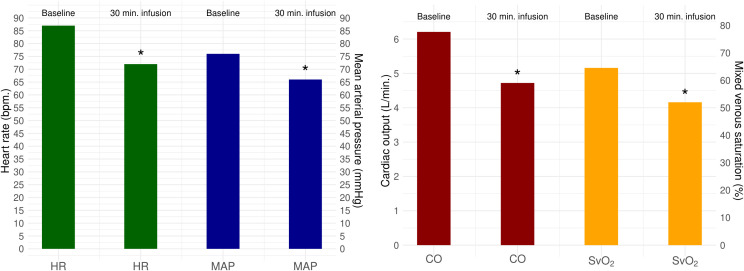
Change in mean arterial pressure, heart rate, cardiac output and mixed venous saturation after 30 minutes of amiodarone infusion compared to baseline values. Changes after 30 minutes of amiodarone infusion compared to baseline values, N = 25. Y-axis of plot to the left with mean arterial blood pressure and heart rate respectively. Y-axis of plot to the right with cardiac output and mixed venous saturation. Significance of 30 min. infusion of amiodarone compared to baseline values: * P ≤ 0.001. HR: Heart rate (bpm), bpm: beats per minute, MAP: Mean arterial pressure (mmHg), CO: Cardiac output (L/min), SvO2: Mixed venous saturation (%).

**Fig 2:**
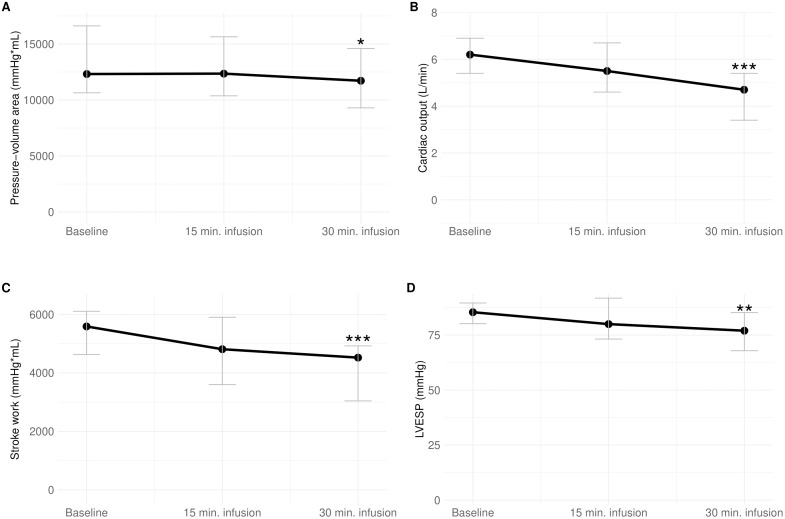
Plot of changes in pressure-volume area, cardiac output, stroke work, and left ventricular end-systolic pressure from baseline to 30 minutes infusion of amiodarone. Plot of median with interquartile range of **A)** Pressure-volume area, **B)** Cardiac output, **C)** Stroke work, and **D)** Left ventricular end-systolic pressure (LVESP) from baseline to 30 minutes infusion of amiodarone. Significance compared to baseline values: * P < 0.05, ** P < 0.01, and *** P < 0.001.

Twenty-five pigs received a 30-minute infusion of amiodarone which led to a significant reduction in systolic blood pressure (SBP) and mean arterial pressure (MAP) (Table 1). After 30 minutes infusion of amiodarone, CO decreased with 24% from baseline (from 6.2 (5.4; 6.9) L/min. to 4.7 (3.4; 5.4) L/min., p < 0.001) and MAP decreased with 13% (76 (72; 83) mmHg to 66 (60; 73) mmHg, p = 0.001) (Fig 1 and 2), without any significant change in systemic vascular resistance (SVR). The decrease in CO was attributed to reduction in both heart rate and stroke volume (Table 2). Concomitantly SvO₂ declined from 65% (55; 72) to 52% (39; 68), p < 0.001 (Fig 1 and Table 1).

**Table 1:** Hemodynamic parameters during infusion of amiodarone.

N = 25	Baseline	30 min. infusion	P-value
**Systolic blood pressure (mmHg)**	103 (97; 108)	91 (86; 100)	<0.001
**Diastolic blood pressure (mmHg)**	60 (53; 68)	51 (47; 58)	0.002
**MAP (mmHg)**	76 (72; 83)	66 (60; 73)	0.001
**CVP (mmHg)**	13 (10; 14)	12 (10; 17)	0.001
SVR (dynes*sec*cm ⁻ ⁵)	797 (730; 1188)	940 (735; 1189)	ns
**Heart rate (bpm)**	87 (79; 99)	72 (62; 81)	<0.001
**SvO2 (% saturation)**	65 (55; 72)	52 (39; 68)	<0.001
**PA systolic pressure (mmHg)**	35 (27; 35)	36 (31; 39)	0.004
**PA diastolic pressure (mmHg)**	13 (9; 20)	15 (11; 20)	ns
**PA mean pressure (mmHg)**	23 (19; 26)	26 (23; 28)	0.005
PVR (dynes*sec*cm ⁻ ⁵)	111 (76; 184)	197 (139; 254)	0.008
**PAPi**	1.4 (1; 2.1)	1.5 (1; 2)	ns

Data is presented as median (interquartile range) and analysed with a Wilcoxon signed rank test.MAP: Mean arterial pressure, CVP: Central venous pressure, SVR: Systemic vascular resistance, bmp: Beats per minute, SvO_2_: Mixed venous saturation, PA: Pulmonary artery, PVR: Pulmonary Vascular Resistance, PAPi: Pulmonary Artery Pulsatility Index. Ns: non-significant.

**Table 2:** Left ventricular conductance catheter derived parameters during infusion of amiodarone.

N = 25	Baseline	15 min. infusion	30 min. infusion	P-value
**CO (L/min)**	6.2 (5.4; 6.9)	5.5 (4.6; 6.7)	4.7 (3.4; 5.4)	<0.001
**PVA (mmHg/mL) x 10** ^**3**^	12.3 (10.6; 16.6)	12.4 (10.4; 15.6)	11.7 (9.3; 14.6)	0.01
PE (mmHg * mL) x 10 ^**3**^	6.8 (5.9; 10.6)	7.5 (5.9; 10.1)	7.8 (5.4; 9.8)	ns
Stroke work (mmHg*mL) x 10 ^**3**^	5.6 (4.6; 6.1)	4.8 (3.6; 5.9)	4.5 (3.0; 4.9)	<0.001
TCW (PVA*HR*10^**−3**^)	1186 (894; 1532)	1030 (769; 1275)	821 (597; 1125)	<0.001
**LVESP (mmHg)**	85 (80; 90)	80 (73; 92)	77 (68; 85)	0.002
**LVEDP (mmHg)**	13 (11; 17)	15 (12; 17)	15 (13; 17)	ns
**LVESV (mL)**	75 (62; 112)	101 (72; 109)	106 (79; 131)	<0.001
**LVEDV (mL)**	144 (132; 169)	155 (139; 183)	160 (134; 184)	ns
**Stroke volume (mL)**	69 (64; 79)	71 (52; 79)	64 (48; 73)	0.004
**LVEF (%)**	44 (39; 54)	41 (34; 45)	38 (34; 42)	<0.001
**EA (mmHg/mL)**	1.19 (1.08; 1.37)	1.20 (0.98; 1.43)	1.25 (1.03; 1.42)	ns
**EES (mmHg/mL)**	0.54 (0.33; 0.63)	0.47 (0.30; 0.57)	0.43 (0.29; 0.51)	<0.001
**EA/EES ratio**	2.45 (2.01; 3.53)	2.90 (2.21; 4.14)	3.26 (2.55; 4.30)	<0.001
**dP/dt max (mmHg/s)**	1280 (1131; 1555)	1164 (932; 1301)	993 (779; 1067)	<0.001
**Cardiac efficiency**	0.4 (0.4; 0.5)	0.4 (0.3; 0.4)	0.4 (0.3; 0.4)	<0.001

Data are presented as median (interquartile range) and analysed with a Wilcoxon signed rank test.CO: Cardiac output, PVA: Pressure-volume area, PE: Potential energy, TCW: Total cardiac work, LVESP: Left ventricular end-systolic pressure, LVEDP: Left ventricular end-diastolic pressure, LVESV: Left ventricular end-systolic volume, LVEDV: Left ventricular end-diastolic volume, LVEF: Left ventricular ejection fraction, EA: arterial elastance, EES: end-systolic elastance, VA-coupling: ventricular-arterial coupling, dP/dt max: maximal rate of rise of left ventricular pressure. Ns: non-significant

**Fig 3:**
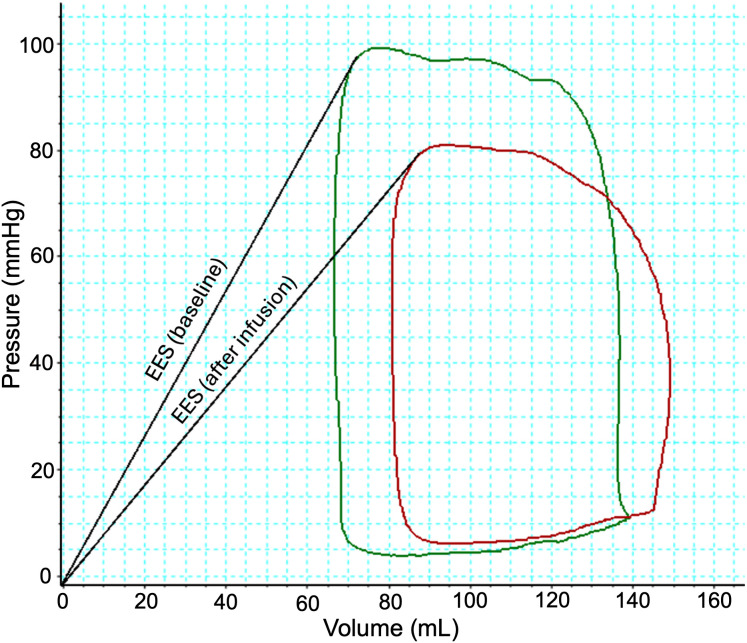
Pressure-volume area at baseline and after 30-minute infusion of amiodarone. Example of pressure-volume loop changes in the left ventricle from baseline (green) to the end of a 30 minutes of amiodarone infusion (red). End-systolic elastance (EES) is shown for both pressure-volume loops with V0 at 0 mL‌‌.

During amiodarone infusion, LV end-systolic pressure (LVESP) decreased from 85 (80; 90) mmHg to 77 (68; 85) mmHg (p = 0.002) (Fig 2, Table 2), while LV end-systolic volume increased from 75 (62; 112) mL to 106 (79; 131) mL (p < 0.001). The maximal rate of pressure rise in the LV (dP/dt max) decreased by 22% (p < 0.001), and end-systolic elastance (EES) decreased significantly too (p < 0.001) (Fig 3).

Arterial elastance (EA) remained unchanged, however, the significant reduction in (EES) led to a marked increase in the EA/EES ratio from 2.45 (2.01;3.53) to 3.26 (2.55; 4.30) (p < 0.001), Table 2.

A significant decrease in stroke work (5.6 (4.6; 6.1) mmHg*mL*10^3^ to 4.5 (3.0; 4.9) mmHg*mL*10^3^, p < 0.001) and pressure volume area (PVA) (12.3 (10.6; 16.6) mmHg*mL*10^3^ to 11.7 (9.3; 14.6) mmHg*mL*10^3^, p = 0.01) were observed (Fig 2 and 3, Table 2) indicating a shift in the energetic efficiency of LV contractility. This was accompanied by an increase in potential energy which did not reach statistical significance.

PA pressures remained unchanged during amiodarone infusion, while an increase in pulmonary vascular resistance (PVR) was observed.

### Organ perfusion

Renal venous pressure remained unchanged (16 (13;18) mmHg to 15 (13;20) mmHg, p = 0.29); however, the renal venous perfusion pressure decreased significantly by 23.3% from 61 (58; 68) mmHg to 47 (41; 54) mmHg (p = 0.004). Carotid artery blood flow decreased significantly by 30% from 269 (230; 361) mL/min to 204 (148; 256) mL/min, p = 0.001.

## Discussion

This translational animal study demonstrates that a bolus amiodarone infusion in anesthetized, healthy female pigs in stable sinus rhythm significantly reduces CO and blood pressure. The reduction in CO was attributable to concomitantly decreased heart rate and stroke volume, due to impaired LV contractility, as evidenced by reduction in EES and dP/dt max. These findings highlight the potential immediate negative inotropic effects on the myocardium associated with bolus amiodarone infusion. Although, the observed changes showed a clear temporal association with drug administration, a causal relationship cannot be established with certainty in the absence of a control group.

Several mechanisms may explain the observed direct cardiac effects of amiodarone. Due to known calcium channels blockage, amiodarone infusion results in decreased intracellular calcium availability during systole, thereby depressing myocardial contractility [1]. Sodium channel inhibition can lead to impairment of cardiac contractility due to reduced cytosolic free calcium concentrations mediated by the Na^+^/Ca^2+^ exchanger, which may result in decreased dP/dt max [17]. Such negative inotropic effects have been demonstrated both in healthy animal models [9,10], and in patients with compromised ventricular function [1]. In our study, we observed a marked decrease in CO, SvO_2_ and EES, indicating reduced myocardial performance. However, the decline in arterial blood pressure, both systolic and MAP, was relatively modest in comparison to CO, relating to a combined reduction in heart rate and stroke volume. EES was significantly reduced, which may also relate to changes in heart rate and loading condition. SVR did not change significantly, indicating that the decrease in blood pressure was primarily driven by the reduced CO, rather than systemic vasodilation. Thus, this reduction in CO and SvO₂ may go unnoticed in the clinical setting if monitoring is limited to blood pressure alone, given the relatively modest decrease in MAP. Our study extends these findings by applying PVL analysis, which enables load-independent assessment of contractility through EES, and thereby offers a more precise quantification of left ventricular mechanics. We demonstrate that the reduction in CO is partly driven by an acute deterioration in LV contractility (dP/dT, EES).

Previous experimental and clinical studies have examined hemodynamic effects of amiodarone. In rabbits with sinus rhythm treated with an amiodarone infusion, Lessa et al. reported a greater decrease in MAP, a reduction in dP/dt-max and CO, and, in contrast to our findings, a reduction in SVR [9]. It should be noted, however, that their calculation of SVR was based on MAP divided by mean aortic flow, without accounting for central venous pressure (CVP) [9], whereas our study applied the standard formula (S1 Table). This methodological difference may partly explain the discrepancy between the results. Salgado et al. demonstrated in conscious rats that a 50 mg/kg intravenous amiodarone dose led to a negative inotropic effect by marked bradycardia and significant reduction in in dP/dt-max, while a dose of 25 mg/kg caused only mild hypotension without affecting contractility, relaxation, or heart rate [18]. However, both studies concluded that despite a significant cardiovascular depression and cardio depressant contractility, amiodarone did not cause LV dysfunction [9,18].

Cushing et al. described negative inotropic effect of amiodarone with a decrease in dP/dt max, attributing hypotension to a combined negative inotropic and vasodilatory mechanism [10]. However, their calculation of systemic vascular resistance (SVR) was based solely on CO and MAP, which is not sufficient to accurately determine SVR. This methodological limitation may contribute to the reported findings and could represent a potential explanation for the observed differences compared with other studies. Consequently, these results should be interpreted with caution. Also, since dP/dt max is load-dependent, it can be challenging to distinguish whether a decrease reflects intrinsic myocardial dysfunction or changes in loading conditions. Thus, including PVL analysis will provide a more comprehensive insight into LV mechanics.

From a clinical perspective, our findings are particularly relevant in the setting of hemodynamically stable patients. Understanding these intrinsic myocardial effects is important, since amiodarone is also administered prophylactically in patients in sinus rhythm, for example in coronary artery bypass grafting patients in sinus rhythm [19]. Cheung et al demonstrated in a randomised trial with coronary artery bypass grafting patients, that a bolus infusion of 150 mg of amiodarone intravenously over 15 minutes led to a significant decrease in arterial blood pressure and heart rate, requiring vasopressor treatment in 20% of the patients [20].

In a study in ischaemic heart disease patients in sinus rhythm, Bopp et al. observed little effect on heart rate, vascular resistance but a 12% decrease in LV work after a rapid 1-minute amiodarone bolus infusion [21]. This is not in line with our findings, however given that the studies have also used an abandoned calculation of SVR with MAP/CO, which does not include the venous component, it can be misleading. CVP fell significantly during the amiodaron infusion, therefore the decrease in MAP and CVP outbalances the decrease in CO leading to unchanged SVR.

The impact of amiodarone is possibly more pronounced in patients with reduced ejection fraction as demonstrated by Kosinski et al, reporting a 20% decrease in cardiac index if LVEF was below 35% [22]. In a dose-ranging study with amiodarone double-blinded, randomised into three groups, the efficacy of amiodaron in terms of VT event free duration increased with increasing amiodarone doses (125 mg, 500 mg or 1000 mg over 24 hours), but interestingly the occurrence of hypotension was equal across the increasing doses [3]. Intravenous amiodarone has quite complex pharmacokinetics. Peak serum concentrations after 15-minute infusions in healthy volunteers range from 5 to 41 mg/L, and peak levels after 150 mg of supplemental infusions in patients with VT/VF range between 7 and 26 mg/L. Because the drug rapidly distribute into the tissue, serum concentrations decline to 10% of peak values within 30–45 minutes after stopping the infusion [23]. This underscores the significance of recognizing amiodarone’s direct cardiac effects across clinical contexts and highlight the importance of close hemodynamic monitoring in patients receiving amiodarone infusion, allowing for timely management with vasopressors or inotropes if needed to maintain sufficient end-organ perfusion.

The strengths of this study include the use of extensive meticulous hemodynamic monitoring, and a standardized, rhythm-stable animal model. Furthermore, our sample size exceeds that of previous translational animal studies on the effect of amiodarone infusion. However, several limitations should be taken into consideration. First, anaesthesia was required for instrumentation in this animal model and may have influenced vascular tone, autonomic regulation, and myocardial contractility. In addition, anaesthesia prior to and during the experimental protocol may itself contribute to time-dependent hemodynamic changes. Because no vehicle-control infusion was performed, an interaction between anaesthesia-related effects and amiodarone administration cannot be fully excluded. Therefore, the magnitude and specificity of the observed hemodynamic effects should be interpreted cautiously when extrapolating to awake conditions or clinical settings. However, the same hemodynamic changes with reduced heart rate and ventricular contractility as well as lower cardiac output has been observed across three different species (rabbit, rats and now pigs), indicating a consistent response in hemodynamics [9,18]. Second, the administered dose (300 mg over 30 minutes) reflects a commonly used intravenous loading regimens in clinical practice and is consistent with recommendation from European and national guidelines [5,24]. However, the observed hemodynamic effects may not be generalizable across different amiodarone doses and rates of infusion. Third, the absence of a placebo or time-control group limits the ability to definitively distinguish drug-related effects from potential time-dependent changes related to prolonged anaesthesia or instrumentation. Although hemodynamic parameters appeared stable prior to amiodarone infusion and the observed changes showed a clear temporal association with drug administration, a causal relationship cannot be established with certainty in the absence of a control condition. Future studies including a vehicle-control arm will therefore be necessary to confirm the specificity and magnitude of the observed effects.

As the primary aim of the study was to evaluate hemodynamic and cardiac performance, underlying biological and molecular mechanisms of amiodarone-induced changes were not investigated; further studies are therefore necessary to explore the associated neurohumoral responses. Furthermore, the immediate effect of an amiodarone bolus was observed within the first 30 minutes, however due to the restricted observation duration, we cannot defer if changes were completely reversible, which should be explored in future studies.

## Conclusion

In this translational animal study, a 30-minute infusion of 300 mg amiodarone intravenously in anesthetized pigs with stable sinus rhythm significantly lowered CO due to reduced heart rate and impaired LV contractility. Arterial blood pressure was only modestly reduced, SVR and EA remained unchanged, resulting in ineffective VA-coupling.

## Supporting information

S1 Fig Supplementary Figure 1. Overview of instrumentation in pigs.
(PDF)

S1 Table Overview of parameters derived or calculated from the conductance catheter. SBP: Systolic blood pressure, DBP: Diastolic blood pressure, MAP: Mean arterial pressure, CVP: Central venous pressure, SVR: Systemic vascular resistance, bpm: Beats per minute, SvO_2_: Mixed venous saturation, ECG: Electrocardiogram, PA: Pulmonary artery, PVR: Pulmonary Vascular Resistance, PAPi: Pulmonary Artery Pulsatility Index, CO: Cardiac output, PVA: Pressure-volume area, PE: Potential energy, TCW: Total cardiac work, LVESP: Left ventricular end-systolic pressure, LVEDP: Left ventricular end-diastolic pressure, LVESV: Left ventricular end-systolic volume, LVEDV: Left ventricular end-diastolic volume, LVEF: Left ventricle ejection fraction, EA: arterial elastance, EES: end-systolic elastance, VA-coupling: ventricular-arterial coupling, dP/dt max: maximal rate of rise of left ventricular pressure.
(DOCX)

S1 File Dataset. The dataset underlying the findings of this study.
(PDF)
