# Supplementary material for: Hemodynamic effects of prophylactic amiodarone assessed by pressure–volume analysis in anesthetized female pigs in sinus rhythm—An exploratory study
Source: PLoS One. 2026 May 22;21(5):e0349600. doi: 10.1371/journal.pone.0349600 (PMC13196969; doi:10.1371/journal.pone.0349600)
Supplement: S1 Fig — Overview of instrumentation in pigs. (PDF) [file pone.0349600.s001.pdf]

ID	time	Pulse	Bpsys	Bpdia	MAP	PAPsys	PAPdia	MPAP	CVP	ETCO2	CO	CI	SvO2	Carotid flov	PI	Renal vein p	Na amount	CO (mL/mi	Ped (mmHg
1	1	94	99	52	68	32	26	28	7	5.8	7.2	3.9	70	446	1.9	7	0.02	7069	9.082
1	3	78	95	51	66	35	30	32	11	5.0	6.8	3.7	50	369	2.4	8	0.02	6777	12.01
2	1	103	107	67	82	26	19	22	5	7.0	6.2	3.4	72	385	1.1			6502	6.567
2	3	90	104	63	79	31	20	26	7	5.9	6.2	3.4	70	275	1.6			6064	10.77
3	1	81	133	77	95	25	15	20			5.9	3.1	74					5083	11.14
3	3	72	117	61	78	28	14	19	10	5.7	6.5	3.4	68	330		15		5013	14.41
4	1	61	91	53	67	38	15	29	10	5.5	6.2			380		8		6058	12.36
4	3	57	99	66	82	39	14	27	10	5.1	6.2		86	406		9		4722	14.51
6	1		140	76	96	27	10	18	14	4.4			77	362	1.1			5440	12.69
6	3	62	116	53	71	30	23	28	17	4.0	5.5	2.9	71	250	3.3	17		2935	15.87
7	1	90	96	52	67	28	9	18	9	5.5	8.5	4.5	85	413	2.3			7755	17.07
7	3	80	102	64	80	35	10	23	8	6.1	8.2	4.3	77	383	2.0			5217	17.49
8	1	107	84	49	62	20	10	15	5	5.2	6.6	3.6	65	232	2.4			6135	9.213
8	3	86	85	54	67	25	17	21	8		5.0	2.7	52					4116	11.82
9	1	78	85	52	62	35	11	23	13					235	1.9			4889	19.71
9	3	61	72	41	53	42	11	24	15	5.5	4.0	2.0	39	107	3.5	15		3435	17.49
10	1	79	102	60	77	34	5	18	10	5.8	3.5		53	250		10		3036	19.45
10	3	62	91	50	65	31	6	18	11	4.9	4.0	2.1	52	165		13		1659	18.17
12	1	85	101	67	82	38	24	30	14	5.9			71	334	1.7			3538	13.28
12	3	59	92	58	71	41	18	27	19	5.7	3.9		45	161	1.7	34		3324	12.32
14	1	90	99	59	76	27	20	23	9	5.9	7.3	3.8	71	303	2.4	20		6725	9.037
14	3	81	88	50	65	33	23	28	10	5.4	7.2	3.6	62			23		5188	11.78
15	1	87	108	74	87	35	6	19	13	5.4	7.0	3.7	54	168		13		6754	12.73
15	3	77	83	50	60	34	7	19	12	4.2	6.2	3.2	48	148		14		7101	13.33
16	1	104	90	67	76	20	8	15	11	6.0	6.1	3.2	84	269	2.5	7		8396	5.743
16	3	70	86	57	69	26	8	17	11	5.5	5.5	2.9	74			9		5584	13.63
**18**	**1**																	8570	12.55
**18**	**3**																	4224	14.18
19	1	84	79	46	59	13	9	11	14	6.5	6.3	3.3	67	257		18		6447	16.58
19	3	61	79	41	54	39	15	29	12	6.1	4.4	2.3	44	204		13		5348	13.83
**20**	**1**																	7185	17.58
20	3	92	96	51	67	33	20	27	11	6.5	6.2	4.6	53	311, 0,7	0.7	15		5369	18.03
**21**	**1**																	6992	18.24
**21**	**3**																	3228	15.47
24	1	101	107	71	84	38	32	35	21	6.5	6.7	3.5	64	230	2.8	20		8365	16.41
24	3	85	92	62	73	40	32	37	22	6.2	5.3	2.8	59	167	3.2	19		6687	17.71
26	1	67	97	58	73	36	11	22	17	7.3	5.1	2.8	52	164	4.4			5848	16.38
26	3	54	88	42	56	50	12	23	20	6.7	4.5	2.4	36	229	3.9			4661	14.82
27	1	94	110	60	77	35	11	22	15	7.4	7.7	4.1	60	361	1.3	14		6917	13.09
27	3	85	86	48	59	36	13	24	20	7.8	6.4	3.5	34	244	3.5	25		3404	12.62
28	1	89	97	60	73	33	23	28	17	7.3	5.5	2.9	42	290	1.7	19		5911	19.83
28	3	79	87	55	66	37	16	26	19	7.1	3.8	2.1	21	115	1.2	21		3855	18.02
**30**	**1**																	5468	15.72
**30**	**3**																	2091	16.05
33	1	79	123	90	105	44	28	36	15	6.2	6.8	3.7	42	189	1.8	18		4866	17.2
33	3	62	83	47	59	47	29	39	17	5.5	3.6	1.8	21	145	3.1	20		3575	15.55
**34**	**1**																	6962	13.4
**34**	**3**																	3339	18.03
35	1	85	102	60	75	35	15	24	14	8.6	5.7	3.2	60	382	1.7	17		4169	15.22

35	3	67	87	50	61	28	27	33	18	5.8	4.8		2.7		34	256	2.5		20	2894	16.88
37	1	104	104	54	70	35	20	24	12	6.5	9.4		5.1		67	276	2.3		18	7995	12.71
37	3	81	93	46	61	37	25	30	12	6.5	7.2		3.9		47				14	6107	15.24
38	1	96.97	108	49	73	39	4	20	7	6.9		6.5		3.5	58	157	0.1		13	6208	11.97
38	3	72.1037	99.9752	41.5754	61.9203	33.9741	5.67855	17.9939	9.59136	6.0	6.5		3.5		41	298	2.3		11	3220	15.45
39	1	103.364	105.32	50.3466	74.1589	33.2193	20.0797	26.0318	5.91262	7.4	9.1		4.7		77	399	1.7		15	3220	5.162
39	3	82.7886	104.009	55.0568	78.3926	38.7993	20.3441	30.1267	9.38964	7.3	8.9		4.7		74	450		2	18	7511	8.028
40	1	71.1037	105.801	58.6162	80.563	35.1638	8.89893	23.6547	13.4247	7.6	6.5		3.3		53	202	1.5		17	6840	17.86
40	3	61.4892	82.7756	42.1098	57.9481	35.9975	7.74602	23.993	15.4669	6.1	5.1		2.6		27	221	1.5		15	4806	12.57
41	1	74.722	132.036	90.2781	109.854	33.888	22.8386	25.9486	11.6838	7.7	6.9		3.7		57	199		1	13	6195	8.983
41	3	70.1756	120.126	82.5759	99.9361	37.0254	13.3922	28.1784	11.9901	6.1	5.6		3.0		53	138	1.3		13	4374	8.33

ID	time	SW (mmHg	CO (mL/mi	SV (mL)	Vmax (mL)	Vmin (mL)	Ves (mL)	Ved (mL)	Pmax (mmH	Pmin (mmH	Pmean (mn	Pdev (mmH	Pes (mmHg	Ped (mmHg	HR (bpm)	EF (%)	Ea (mmHg/	PowMax (m	dP/dt max	dP/dt min (	dV/dt max	dV/dt min (	P@dV/dt m	P@dP/dt m	V@dP/dt m	V@dP/dt m	Tau (ms)	ESPVR (mm	Piso (mmHg		V0linear	V0exponen
1	1	6041	7069	73.8	163.8	81.03	86.86	160.6	89.63	3.441	37.3	86.19	82.81	9.082	95.79	47.25	1.124	5838	1237	-1507	923.7	-608.9	3.587	47.34	156.2	82.82	28.38				-77.3	
1	2	5268	5929	70.22	164	85.93	90.09	159.8	84.29	3.868	33.72	80.42	76.35	11.13	84.43	44.8	1.088	6160	1078	-1289	727.8	-553.8	3.92	44.67	156.8	86.93	-4016	-9284	-1.342	31.24	-77.3	
1	3	6326	6777	86.22	182.4	90.93	97.82	165.8	86.38	5.451	35.39	80.93	80.17	12.01	78.6	48.18	0.9306	7162	1066	-1279	782.7	-668.1	5.663	47.06	178.9	92.67	1444	-4883	4.55	35.36	-77.3	
2	1	5354	6502	61.18	188.6	117.4	120.7	186.6	93.92	1.798	42.67	92.12	88.16	6.567	106.3	33.83	1.445	8637	1420	-1541	1029	-560.2	1.865	47.66	180.7	118.9	1844	-3510	2.931	29.23	-86.2	-42
2	2	5901	6636	67.27	200.9	123.2	125.4	194.8	97.1	4.011	45.24	93.08	93.48	9.914	98.67	34.47	1.392	1.94E+04	1304	-1454	778.3	-1032	4.542	50.47	195	126.6	3.24E+04	2.65E+04	0.1825	35.61	-86.2	-42
2	3	5605	6064	67.67	212.4	135.2	137.8	201.8	92.76	4.722	41.55	88.03	89.27	10.77	89.64	33.06	1.321	1.49E+04	1138	-1326	704.8	-939.1	4.927	47.33	204.6	137.1	2.53E+04	1.97E+04	0.2218	38.17	-86.2	-42
3	1	5616	5083	64.19	177.7	101.9	106.6	159.9	101.9	5.727	46.4	96.18	98.34	11.14	79.18	38.29	1.534	9604	1270	-2150	640	-425.2	9.022	49.43	167.7	102.8	53.31	-5562	105.4	40.98	-131.769	-77
3	2	6319	5757	72.41	184.9	102.3	108.2	163.6	104.1	7.209	49.55	96.91	101.1	12.75	79.51	41.39	1.4	9571	1302	-2180	579.3	-432.1	14.02	53.12	174.9	102.9	54.09	-6265	117	44.81	-131.769	-77
3	3	5350	5013	68.49	184.4	108.1	115.1	163	96.74	7.908	44.3	88.84	93.83	14.41	73.21	38.53	1.373	1.08E+04	1136	-2144	587.5	-409.3	16.92	48.15	177.7	108.8	57.53	-5293	93.15	47.57	-131.769	-77
4	1	6124	6058	69.45	141.2	66.21	70.65	139.6	100.3	4.969	46.96	95.29	97.57	12.36	87.22	50.62	1.406	3470	1770	-2321	525.9	-528.7	5.624	55.86	137.2	66.79	35.32	-6089	173.8	38.37	-14	-25.8
4	2	4657	5291	65.13	145.6	74.23	78.98	144	83.97	5.849	38.42	78.12	80.04	13.06	81.24	46.17	1.23	3839	1307	-1451	554.5	-482.2	6.332	46.39	141.1	74.74	39.49	-4618	118.2	42.73	-14	-25.8
4	3	4431	4722	65.24	149	78.84	83.71	146.6	82.33	6.981	36.35	75.35	78.21	14.51	72.35	44.74	1.201	4354	1097	-1289	447.9	-440.8	8.29	47.19	145.8	79.54	41.85	-4390	106.1	47.76	-14	-25.8
6	1	5394	5440	69.43	129.4	47.24	56.12	125.8	88.16	3.776	38.48	84.38	83.71	12.69	78.35	57.61	1.208	6690	1158	-1995	1187	-446.4	4.648	40.64	120.5	51.36	8737	3342	0.6175	34.93	-95.4	-16.2
6	2	3598	3111	46.29	155.8	102.6	106.9	151.6	95.97	6.261	39.21	89.71	94.8	15.39	67.15	30.69	2.057	8729	990.3	-1924	549.6	-401.5	10.13	49.38	150.8	104.2	8751	5153	0.4109	42.54	-95.4	-16.2
6	3	3696	2935	48.12	150.1	86.05	87.52	143.8	89.64	6.721	35.7	82.92	87.71	15.87	60.85	35.5	1.834	1.45E+04	881.5	-1839	622	-452	8.578	44.11	135.5	88.41	2.18E+04	1.81E+04	0.1699	43.43	-95.4	-16.2
7	1	6301	7755	84.54	201.7	101.3	103.6	194.1	87.41	7.846	42.9	79.57	80.34	17.07	91.73	43.83	0.9507	2.19E+04	1430	-1744	840.9	-637.1	8.252	52.31	192.9	109.5	1.04E+04	4070	0.6076	38.1	-211	-77.7
7	2	7474	7544	83.5	196.9	101.1	103.1	184.2	103.2	7.962	50.43	95.28	100.2	16.16	90.31	43.76	1.201	1.94E+04	1809	-2133	868.4	-698.1	8.48	63.95	190.8	107.1	51.54	-7423	145.1	35.48	-211	-77.7
7	3	4922	5217	68.44	193.9	110.1	113.1	181.6	87.01	8.912	40	78.1	83.15	17.49	76.24	37.08	1.218	1.72E+04	1158	-1541	708.5	-561.2	9.299	49.33	184.5	116.3	2.64E+04	2.15E+04	0.1867	44.3	-211	-77.7
8	1	4228	6135	57.26	173.4	108.9	114.5	166.2	81.17	3.364	44.94	77.81	77.82	9.213	107.2	34.24	1.368	5383	1385	-1335	683.8	-443.7	4.837	45.7	167.2	110.1	6664	2435	0.6344	36.64	-144.05	-48.5
8	2	3549	4792	51.31	165.7	105.1	116.8	155.4	79.06	4.517	42.62	74.54	77.13	11.68	93.39	32.15	1.505	5748	1227	-1335	596.8	-330.5	9.653	46.83	159.6	108.7	58.41	-3490	60.83	40.72	-144.05	-48.5
8	3	3021	4116	47.73	120.5	71.03	75.09	115.4	77.42	6.927	41.26	70.49	71.36	11.82	86.21	39.87	1.502	4534	1016	-1070	403	-319.5	7.468	42.25	119.8	71.8	37.55	-2983	81.04	53.5	-144.05	-48.5
9	1	4509	4889	63.68	199.8	123.2	126.1	193.8	88.17	12.4	43.92	75.76	86.02	19.71	76.79	33.05	1.355	1.51E+04	950.8	-1571	535.7	-446.7	15.55	51.89	192.6	127.4	2.31E+05	2.26E+05	0.01953	60.31	-163	-172.4
9	2	3276	3723	51.99	187.9	123.2	127.4	182.9	79.19	11.61	38.09	67.59	76.49	18.52	71.64	29.01	1.477	1.11E+04	771.9	-1136	450.4	-389.6	16.79	46.45	179.1	126.7	5.37E+04	5.05E+04	0.06099	65.83	-163	-172.4
9	3	2906	3435	55.44	189.5	124.8	132.6	184.4	69.81	11.48	31.5	58.33	66.61	17.49	62	30.3	1.205	8367	611.2	-908.6	450.1	-369.9	14.81	38.73	182.9	128.3	2.99E+04	2.70E+04	0.09707	72.1	-163	-172.4
10	1	3027	3036	38.24	104.5	59.5	61.9	103	93.68	8.115	42.11	85.56	89.29	19.45	79.41	38.72	2.336	4238	1061	-1746	377.5	-256.1	9.143	55.62	98.75	59.89	8.01E+04	7.71E+04	0.03778	39.77	-80.67	-48.35
10	2	2247	2047	30.4	108.7	70.46	72.42	107.2	92.35	9.944	38.45	82.41	89.5	19.87	67.35	29.94	2.95	4466	1039	-1658	287	-229.8	10.68	54.99	101.5	71.1	7.07E+04	6.84E+04	0.03179	45.88	-80.67	-48.35
10	3	1890	1659	29.38	113.8	76.25	78.31	111.9	83.58	9.549	32.38	74.03	80.97	18.17	56.5	27.32	2.764	5215	825.3	-1362	281.4	-203.3	11.06	42.59	107.5	77.74	3.90E+04	3.71E+04	0.04846	47.16	-80.67	-48.35
12	1	3848	3538	62.39	123.7	55.1	66.27	111.3	77.35	6.535	29.86	70.81	73	13.28	56.71	51.05	1.174	6466	718.7	-1347	523.2	-415.5	8.803	35.33	122.2	59.53	1.19E+04	8051	0.3235	49.05	-68.3	-42.41
12	2	4651	4592	74.67	171.8	92.28	102.3	167.2	77.09	6.334	30.83	70.75	73.2	13.71	61.51	44.11	0.9822	6066	741.8	-1335	662.6	-497.9	7.814	35.45	169.3	93.97	1.31E+04	8477	0.3543	47.11	-68.3	-42.41
12	3	3631	3324	56.6	244.5	168.1	186.7	230.5	77.24	5.95	30.37	71.29	74.68	12.32	58.71	24.18	1.342	1.73E+04	838.8	-1256	749.2	-682.7	12.14	31.66	233.4	175.9	4.18E+04	3.81E+04	0.0869	45.54	-68.3	-42.41
14	1	5627	6725	74.37	179	98.87	111.9	169	82.62	2.484	40.53	80.14	80.39	9.037	90.44	42.51	1.084	1.34E+04	1609	-1520	837.4	-514.2	2.882	47.35	174.9	102.4	1915	-3712	3.008	33.22	-74.35	-52
14	2	4548	5188	57.55	161.7	86.1	108.8	149.6	87.45	3.904	43.53	83.55	85.24	11.78	90.21	38.61	1.509	2.69E+04	1501	-1661	570.3	-588.1	21.21	48.44	148.8	94.21	-8.64E+04	-9.10E+04	-0.05313	37.91	-74.35	-52
14	3	4548	5188	57.55	161.7	86.1	108.8	149.6	87.45	3.904	43.53	83.55	85.24	11.78	90.21	38.61	1.509	2.69E+04	1501	-1661	570.3	-588.1	21.21	48.44	148.8	94.21	-8.64E+04	-9.10E+04	-0.05313	37.91	-74.35	-52
15	1	6305	6754	77.36	139.5	56.04	58.9	132.6	94.83	6.175	43.82	88.66	91.65	12.73	87.29	57.34	1.187	7700	1168	-1722	846.7	-572.9	6.468	42.37	134.9	57.52	3.93E+04	3.30E+04	0.1605	41.81	-72.32	-82
15	2	5428	6963	83.33	145	49.43	51.21	138.9	76.63	4.85	35.05	71.78	70.42	11.99	83.51	61.12	0.8475	9575	931.6	-1098	767.9	-753.6	6.936	36.78	136.3	52.9	8422	2994	0.6443	43.63	-72.32	-82
15	3	5210	7101	88.62	154.6	53.68	54.85	146.4	72.07	5.262	33.05	66.81	64.3	13.33	80.04	59.25	0.732	1.25E+04	774.4	-946.1	740.6	-796.9	7.232	32.4	149.3	60.14	2.65E+04	2.13E+04	0.1969	45.87	-72.32	-82
16	1	6059	8396	70.14	133.6	59.47	61.73	120.8	95.59	1.476	50.04	94.12	92.86	5.743	119.7	52.7	1.326	1.09E+04	1886	-2139	638.8	-768.6	2.011	54.89	133.1	61.45	7284	1224	0.8324	28.07	-85.7	
16	2	5811	7239	81.42	189	96.37	101.3	187.1	83.39	4.036	37.88	79.36	78.11	12.28	88.9	43.91	0.9615	1.96E+04	1176	-1636	633	-751.9	4.856	44.34	185.4	104.7	7813	2003	0.7437	34.59	-85.7	
16	3	4878	5584	73.17	189.8	103.5	110.5	187.3	79.97	4.811	34.04	75.15	77.04	13.63	76.25	39.35	1.056	2.44E+04	999	-1485	584.3	-585.4	16.8	39.54	185.9	113.7	3.29E+04	2.81E+04	0.1481	38.1	-85.7	
**18**	1	6465	8570	88.44	183.2	77.76	79.23	175.1	83.88	4.972	42.96	78.9	74.65	12.55	96.93	49.53	0.8453	3.00E+04	1204	-1290	788.1	-704.7	11.67	45.21	178.6	88.46	1.60E+04	9567	0.4036	36.99	-114.8	26
**18**	2	5186	6216	74.36	182.7	91.09	92.72	169.4	81.05	5.883	39.21	75.17	73.26	12.51	83.59	41.57	0.9866	2.94E+04	1031	-1181	612.8	-604.3	17.16	46.23	178.8	103.5	1.70E+04	1.18E+04	0.3048	42.73	-114.8	26
**18**	3	3222	4224	59.55	183.5	112.4	131.9	161.9	72.44	7.049	32.85	65.39	65.23	14.18	70.9	32.81	1.101	1.84E+04	756.7	-918.2	734.8	-727.9	15.02	40.22	181.3	116.6	3.48E+04	3.16E+04	0.09259	53.88	-114.8	26
19	1	5350	6447	77	132.2	45.28	50.08	129	81.77	7.133	38.71	74.63	77.99	16.58	83.72	62.22	1.019	7779	1339	-1344	744.8	-615.7	7.816	42.27	123.8	47.39	1.20E+04	6645	0.446	41.17	-74.77	-25
19	2	4687	5900	72.42	125.6	43.64	48.12	121.8	78.63	7.949	36.89	70.68	71.06	15.97	81.5	62.06	0.983	8740	1213	-1104	617.1	-549.7	8.716	45.6	116.6	44.2	3770	-917	1.245	47.66	-74.77	-25
19	3	4609	5348	78.06	124	35.91	38.84	112.7	73.01	7.247	31.07	65.77	62.74	13.83	68.54	67.94	0.8057	8006	1035	-935.2	542.2	-560.3	10.16	39.9	114.8	36.65	3348	-1262	1.382	49.33	-74.77	-25
**20**	1	5497	7185	68.54	146.8	71.16	74.99	143.9	95.42	8.831	51.1	86.59	91.7	17.58	104.8	48.66	1.339	9652	1626	-1810	833.8	-433.3	10.44	54.69	140.8	72.63	9760	4262	0.5632	39.34	-62.3	-82.5
20	2	4809	6801	70.48	162.8	85.81	89.77	155	84.13	8.295	43.21	75.84	77.43	16.57	96.49	44.81	1.099	7574	1258	-1319	663.8	-427.4	9.431	47.26	157.3	86.65	1.93E+04	1.45E+04	0.249	40.91	-62.3	-82.5
20	3	4051	5369	64.18	171.9	101.8	106.2	158.8	82.18	10.62	40.93	71.56	77.89	18.03	83.66	38.17	1.215	8752	993.2	-1266	571.1	-392.2	12.06	45.57	168.1	103.9	564.7	-3486	8.907	49.53	-62.3	-82.5
**21**	1	6302	6992	85.13	172.2	66.43	78.64	169.9	88.26	6.15	40.99	82.11	80.59	18.24	82.13	56.1	0.9483	1.06E+04	1302	-1377	1215	-688.7	6.429	45.04	151.7	66.87	9889	3586	0.6371	38.22	-61.11	
**21**	2	5985	5766	89.8	176.8	65.04	75.75	159.4	82.74	7.76	34.67	74.98	76.04	16.08	64.21	57.05	0.8474	1.14E+04	990	-1127	999	-675	10.23	37.78	157.4	67.79	3613	-2371	1.663	46.05	-61.11	
**21**	3	3265	3228	75.43	193.6	83.66	94.8	184.4	58.73	8.575	23.03	50.15	55.63	15.47	42.81	48.07	0.7379	1.36E+04	575.4	-627.7	1110	-685.3	11.74	32.88	156.9	83.78	1227	-2038	2.666	58.22	-61.11	
24	1	5587	8365	83.37	155.4	59.49	65.94	154.4	79.82	6.232	40.25	73.59	71.82	16.41	100.4	58.14	0.8629	7187	1196	-1526	922	-547.7	7.296	44.9	143.4	59.67	2219	-3369	2.522	38.91	-97.64	-79
24	2	6375	6687	72.51	176.6	98.49	106.9	165.8	109.4	11.84	54.66	97.53	108	17.71	92.24	42.3	1.492	8483	1056	-2356	570.9	-493.3	12.74	55.99	171.4	98.8	4154	-2221	1.54	48.15	-97.64	-79
24	3	6375	6687	72.51	176.6	98.49	106.9	165.8	109.4	11.84	54.66	97.53	108	17.71	92.24	42.3	1.492	8483	1056	-2356	570.9	-493.3	12.74	55.99	171.4	98.8	4154	-2221	1.54	48.15	-97.64	-79
26	1	6105	5848	86.72	151.5	55.15	57.72	132.9	86.15	5.647	34.14	80.5	80.24	16.38	67.45	59.41	0.9282	1.21E+04	1020	-1620	1046	-772.8	6.677	42.67	145.9	57.61	1.75E+04	1.14E+04	0.349	35.95	-74.12	-71
26	2	6105	5848	86.72	151.5	55.15	57.72	132.9	86.15	5.647	34.14	80.5	80.24	16.38	67.45	59.41	0.9282	1.21E+04	1020	-1620	1046	-772.8	6.677	42.67	145.9	57.61	1.75E+04	1.14E+04	0.349	35.95	-74.12	-71
26	3	4795	4661	80.2	153.7	65.07	70.19	130.9	77.02	5.911	28.53	71.11	69.39	14.82	58.12	54.48	0.8712	1.01E+04	788.9	-1181	855.7	-604.5	8.859	36.27	147	67.04	1.29E+04	8052	0.3729	39.64	-74.12	-71
27	1	5920	6917	74.54	145.6	56.71	61.95	142	88.41	3.826	38.57	84.58	85.25	13.09	92.8	54.21	1.147	1.18E+04	1131	-2024	952.7	-698.8	4.87	46.32	137.4	62.2	1.91E+04	1.31E+04	0.3107	31.96	-185.23	-131
27	2	2879	4475	52.04	143.5	64.64	70.82	132.6	65.19	4.141	27.23	61.05	58.53	11.97	86.01	42.47	1.131	1.14E+04	745.4	-1053	1026	-697.4	5.947	32.93	122.4	69.4	8245	5366	0.349	33.51	-185.23	-131
27	3	2189	3404	41.08	143.5	73.51	78.59	133.7	63.82	7.279	26.81	56.54	58.48	12.62	82.87	34.09	1.436	1.66E+04	628.3	-900.6	841.5	-633.1	8.975	36.72	120.7	82.28	-453.7	-2643	-6.519	44.07	-185.23	-131
28	1	4348	5911	66.52	80.44	9.047	9.805	76.36	82.13	9.626	40.55	72.5	68.52	19.83	88.85	84.3	1.035	1.19E+04	882.1	-1284	842.6	-531.4	10.69	41.12	78.88	12.86	1.19E+04	7534	0.366	43.74	-6.7	
28	2	3294	4984	58.69	72.78	9.336	10.4	70.14	74.13	10.1	35.87	64.03	58.47	18.69	84.91	83.41	1.011	8497	674.5	-1039	623.6	-445.8	10.63	36.4	70.34	12.2	4635	1342	0.7098	51.94	-6.7	
28	3	2510	3855	47.56	84.16	30.49	31.78	80.13	71.24	11.84	34.49	59.39	63.94	18.02	81.08	58.13	1.356	1.07E+04	539	-981.4	507.5	-437.6	12.85	31.82	81.79	34.79	1.08E+04	8317	0.2318	63.4	-6.7	
**30**	1	4482	5468	55.88	169.3	88.93	93.85	164.1	87.21	5.167	39.51	82.04	79.01	15.72	97.85	38.01	1.418	2.10E+04	1255	-1526	819.1	-890.7	9.762	50.74	147	92.68	1.06E+04	6112	0.4231	31.43	-65.4	-18
**30**	2	2677	3649	43.12	185.5	116.8	123.6	181.7	78.98	8.297	34.28	70.69	74.75	16.35	84.61	26.74	1.749	2.49E+04	913.7	-1208	764.4	-733.9	10.5	42.9	161.3	118.1	1.24E+04	9725	0.2158	41.52	-65.4	-18
**30**	3	1600	2091	29.13	187.8	129.6	131.9	180.6	75.23	9.264	31	65.97	71.92	16.05	71.9	17.4	2.557	2.54E+04	775	-1057	742.3	-595.5	14.64	35.78	166.9	138.1	1.12E+04	9587	0.1422	46.96	-65.4	-18
33	1	4628	4866	57.82	146.3	71.92	74.15	136.2	92.53	7.003	41.28	85.53	89.55	17.2	84.16	44.24	1.551	1.03E+04	1280	-1876	663.4	-546.7	10.93	56.03	130.7	73.11	-1.63E+04	-2.10E+04	-0.2849	35.76	-60.16	-35
33	2	3965	3557	50.65	164.6	99.13	101.3	157.1	92.03	9.595	37.26	82.44	90.6	17.05	70.27	32.41	1.813	2.05E+04	825.8	-1645	728.9	-627.3	15.75	41.7	156.1	105.6	7959	3993	0.4965	46.13	-60.16	-35
33	3	3044	3575	56.52	166.8	96.4	98.8	160.3	69.91	8.408	28.44	61.5	67.89	15.55	63.26	35.04	1.208	1.93E+04	568.7	-1089	754.3	-668.8	10.23	31.71	161.2	103.5	6634	3591	0.4581	50.62	-60.16	-35
**34**	1	4815	6962	72.92	165.3	80.13	86.48	163.7	79.8	5.593	38.12	74.21	72.77	13.4	95.47	46.78	0.9996	1.87E+04	1183	-1621	807.6	-497.1	6.152	41.84	155.9	87.14	1.11E+04	6247	0.4351	34.43	-135.5	-55
**34**	2	3449	5049	60.91	157.1	88.48	97.89	155	71.88	6.972	32.36	64.91	66.33	14.69	82.87	40.39	1.095	1.21E+04	822	-1553	594.9	-412	7.261	35.27	150.7	95.41	2107	-1342	1.637	32.32	-135.5	-55
**34**	3	2445	3339	46.46	165.5	109.1	117.6	162.6	68.21	8.171	29.3	60.04	65.76	18.03	71.84	29.53	1.428	9011	664.7	-1048	473.4	-357.9	8.64	33.21	157.4	112.9	527.9	-1917	4.857	50.26	-135.5	-55
35	1	3317	4169	50.99	139.9	84.29	88.46	132.1	75.86	5.982	34.61	69.88	71.35	15.22	81.77	37.38	1.403	8202	991.2	-1124	678.9	-460.4	7.178	41.5	136.4	86.98	1.12E+04	7879	0.2962	35.75	-38.9	
35	2	3293	3994	49.07	140.2	84.11	86.95	136.8	78.45	7.514	35.78	70.94	73.34	15.38	81.39	36.32	1.501	8598	944.7	-1186	660.5	-506.6	7.797	38.93	135	87.55	3776	482.5	0.8718	42.95	-38.9	
35	3	2480	2894	43.39	133.7	82.87	87.43	131.5	70.99	9.362	31.28	61.63	66.49	16.88	66.67	34.22	1.535	7190	779	-926.6	414.1	-374.7	10.31	33.57	126.8	85.65	7097	4617	0.3494	57.64	-38.9	
37	1	5997	7995	79.21	174	84.36	85.78	165.1	86.14	4.841	40.04	81.3	81.93	12.71	101	46.76	1.037	2.98E+04	1449	-2407	1050	-930.1	7.45	53.75	169.4	92.31	1.63E+04	1.03E+04	0.3688	30.6	-173.4	-308
37	2	5890	7710	88.14	164.3	67.97	70.12	155	78.71	6.644	35.02	72.07	71.38	14.57	87.46	55.93	0.8106	1.33E+04	1164	-1542	1008	-845	6.937	46.63	157.7	72.3	2.70E+04	2.11E+04	0.2184	41.3	-173.4	-308
37	3	4901	6107	79.29	183.2	95.76	98.78	175.1	75.63	6.905	32.11	68.72	71.29	15.24	77.01	44.11	0.8998	2.23E+04	931.5	-1472	968.1	-792.8	7.236	40.29	179.8	104.6	6946	2045	0.7056	41.59	-173.4	-308
38	1	5085	6208	64.03	172.6	96.93	103	169.3	92.1	2.74	41.79	89.36	88.3	11.97	96.96	38.54	1.379	2.40E+04	1555	-1892	927.7	-1145	2.987	49.87	166.2	102.1	1.20E+04	6913	0.4238	25.34	-52.24	-15
38	2	4822	5175	63.77	170.4	93.82	99.4	166.3	87.89	4.015	37.26	83.88	83.33	16.74	81.27	38.96	1.313	2.46E+04	1301	-1715	837.5	-898.5	4.29	47.55	163.6	99.43	-454.5	-5277	-8.462	30.89	-52.24	-15
38	3	3042	3220	44.33	176.6	120	131.3	173.7	83.2	5.517	34.6	77.68	81.42	15.45	72.64	26.5	1.844	1.30E+04	996.1	-1639	701.8	-682.7	6.722	41.02	167.2	122.1	1.09E+04	7846	0.2794	36.42	-52.24	-15

39	1	6760	8343	80.64	227.8	124.6	131.4	215.7	91.92	0.4266	44.26	91.49	86.85	5.162	103.5	38.75	1.08	1.84E+04	1617	-1742	1049	-1070	0.5279	43.28	208.1	127.3	65.72	-6694	102.9	23.95	-136	-55
39	2	6550	7421	79.45	270.7	178.1	184.3	249.2	95.82	1.355	45.02	94.46	91.82	7.902	93.41	30.44	1.158	1.44E+04	1402	-1867	820.7	-735.2	6.253	43.39	260.9	180.1	2.19E+04	1.53E+04	0.2994	26.94	-136	-55
39	3	7550	7511	92.04	270.6	147	151	224.4	94.23	1.952	40.7	92.28	89.19	8.028	81.6	37.62	0.9721	2.58E+04	1276	-1794	1744	-1076	12.18	43.64	244.6	151.1	75.51	-7475	100.3	30.1	-136	-55
40	1	7485	6840	95.04	229	115.9	121.7	224.2	90.56	5.052	40.5	85.51	85.41	17.86	72	43.56	0.9007	4.13E+04	1246	-1647	1074	-651.7	5.219	49.36	218.1	124.1	4508	-2977	1.67	36.67	-145.4	-52
40	2	5579	6014	85.94	222.8	123.1	133.5	217.3	76.63	4.906	34.13	71.72	72.15	15.4	70.04	40.02	0.8417	3.20E+04	920.2	-1173	827.3	-594.8	9.484	40.43	214.7	132.9	4185	-1394	1.336	38.2	-145.4	-52
40	3	4522	4806	78.19	212.7	123	139.8	194.3	70.86	4.556	29.6	66.31	68.19	12.57	61.48	38.21	0.8757	7644	726.7	-836.5	718.4	-510.4	5.148	34.74	204.6	125.5	441.1	-4081	12.62	39.15	-145.4	-52
41	1	8476	6195	82.78	215.6	116.1	118.7	200.6	114	3.091	47.12	110.9	111.8	8.983	74.87	39.94	1.352	1.39E+05	1564	-2433	1672	-1579	69.76	52.84	207.2	124.5	3601	-4876	2.356	36.4	-221	
41	2	7258	5462	74.42	227.3	137.7	139.8	205.7	110.3	3.964	46.09	106.3	108.8	8.926	73.4	33.83	1.463	1.37E+05	1263	-2118	1657	-1500	58.3	61.32	220	146	2918	-4341	2.49	43.01	-221	
41	3	5723	4374	62.92	250.8	168.6	171.5	230.1	100.9	3.436	40.05	97.47	100.1	8.33	69.52	26.22	1.592	1.32E+05	1067	-1922	1791	-1765	52.15	57.81	240	179.3	2706	-3018	2.12	42.26	-221	
