# Supplementary material for: Hemodynamic effects of prophylactic amiodarone assessed by pressure–volume analysis in anesthetized female pigs in sinus rhythm—An exploratory study
Source: PLoS One. 2026 May 22;21(5):e0349600. doi: 10.1371/journal.pone.0349600 (PMC13196969; doi:10.1371/journal.pone.0349600)
Supplement: S1 Table — SBP: Systolic blood pressure, DBP: Diastolic blood pressure, MAP: Mean arterial pressure, CVP: Central venous pressure, SVR: Systemic vascular resistance, bpm: Beats per minute, SvO2: Mixed venous saturation, ECG: Electrocardiogram, PA: Pulmonary artery, PVR: Pulmonary Vascular Resistance, PAPi: Pulmonary Artery Pulsatility Index, CO: Cardiac output, PVA: Pressure-volume area, PE: Potential energy, TCW: Total cardiac work, LVESP: Left ventricular end-systolic pressure, LVEDP: Left ventricular end-diastolic pressure, LVESV: Left ventricular end-systolic volume, LVEDV: Left ventricular end-diastolic volume, LVEF: Left ventricle ejection fraction, EA: arterial elastance, EES: end-systolic elastance, VA-coupling: ventricular-arterial coupling, dP/dt max: maximal rate of rise of left ventricular pressure. (DOCX) [file pone.0349600.s002.docx]

| **Parameter** | **Derived from or calculation** |
| --- | --- |
| **SBP (mmHg)** | Right carotid artery |
| **DBP (mmHg)** | Right carotid artery |
| **MAP (mmHg)** | Right carotid artery |
| **CVP (mmHg)** | PA catheter |
| **SVR (dynes*sec*cm⁻⁵)** | $SVR= \frac{(MAP-CVP)\cdot80}{CO}$ |
| **Heart rate (beats per min.)** | electrocardiogram electrodes |
| **SvO_2_ (% saturation)** | PA-catheter |
| **PA systolic pressure (mmHg)** | PA-catheter |
| **PA diastolic pressure (mmHg)** | PA-catheter |
| **PA mean pressure (mmHg)** | PA-catheter |
| **PVR** | $PVR= \frac{mean PAP-(Diastolic PAP-2)}{CO}\cdot80$ |
| **PAPi** | $PAPi=\frac{Systolic PAP-Diastolic PAP}{CVP}$ |
| **CO (L/min)** | Conductance catheter |
| **PVA (mmHg*mL)** | $PVA=PE+SW$ |
| **PE (mmHg * mL)** | $PE=0.5\cdot P_{es}\cdot V_{es}-V_{0}$ |
| **Stroke work (mmHg*mL)** | Conductance catheter |
| **TCW (PVA*HR*10^-3^)** | $TCW=PVA\cdot HR\cdot{10}^{-3}$ |
| **LVEDP (mmHg)** | Conductance catheter |
| **LVESP (mmHg)** | Conductance catheter |
| **LVESV (mL)** | Conductance catheter |
| **LVEDV (mL)** | Conductance catheter |
| **Stroke volume (mL)** | Conductance catheter |
| **LVEF (%)** | Conductance catheter |
| **EA (mmHg/mL)** | $EA= \frac{P_{max}-P_{min}}{stroke volume}$ |
| **EES (mmHg/mL)** | $EES= \frac{P_{es}}{V_{es}-V_{o}}$ |
| **VA-coupling** | $VA-coupling=\frac{EA}{EES}$ |
| **dP/dt max (mmHg/s)** | Conductance catheter |
| **Cardiac efficiency** | $Cardiac efficiency=\frac{Stroke work}{PVA}$ |
| **Mean renal venous pressure** | Renal vein catheter |
| **Renal venous perfusion pressure** | $Renal venous perfusion pressure=Mean renal vein pressure-MAP$ |
| **Carotid blood flow** | Doppler flow probe |
